# The substructure of three repetitive DNA regions of *Schistosoma haematobium* group species as a potential marker for species recognition and interbreeding detection

**DOI:** 10.1186/s13071-017-2281-7

**Published:** 2017-08-01

**Authors:** Ibrahim Abbasi, Bonnie L. Webster, Charles H. King, David Rollinson, Joseph Hamburger

**Affiliations:** 1 Department of Microbiology and Molecular Genetics, The Institute for Medical Research Israel-Canada, The Kuvin Centre for the Study of Infectious and Tropical Diseases, The Hebrew University - Hadassah Medical School, The Hebrew University of Jerusalem, 91120 Jerusalem, Israel; 2 Department of Biological Sciences, Faculty of Science and Technology, Al-Quds University, Abu Deis, Palestine; 3 Department of Life Sciences, Parasites and Vectors Division, The Natural History Museum, London, SW7 5BD UK; 4 WHO Collaborating Centre for Schistosome and Snail Identification and Characterisation, London, UK; 5 London Centre for Neglected Tropical Disease Research (LCNTDR), London, UK; 6 Center for Global Health and Diseases and WHO Collaborating Centre for Research and Training for Schistosomiasis Elimination, Case Western Reserve University, School of Medicine, Cleveland, OH USA

**Keywords:** *Schistosoma haematobium*, *Schistosoma bovis*, Hybridisation, DraI, Inter-repeat linkers, Reverse line blot (RLB)

## Abstract

**Background:**

*Schistosoma haematobium* is the causative agent of human urogenital schistosomiasis affecting ~112 million people in Africa and the Middle East. The parasite is transmitted by snails of the genus *Bulinus*, which also transmit other closely related human and animal schistosomes. The accurate discrimination of *S*. *haematobium* from species infecting animals will aid effective control and elimination programs. Previously we have shown the utility of different repetitive nuclear DNA sequences (DraI, sh73bp, and sh77bp) for the identification of *S*. *haematobium-*group species and inter-repeat sequences for discriminating *S*. *haematobium* from *S. bovis*.

**Results:**

In this current study we clarify the structural arrangement and association between the three repetitive sequences (DraI, sh73bp, and sh77bp) in both *S*. *haematobium* and *S. bovis*, with a unique repeat linker being found in *S*. *haematobium* (Sh64bp repeat linker) and in *S. bovis* (Sb30bp repeat linker). Sequence data showed that the 3′-end of the repeat linker was connected to the DraI repetitive sequence array, and at the 5′-end of the repeat linker sh73bp and sh77bp were arranged in an alternating manner. Species-specific oligonucleotides were designed targeting the species-specific repeat linkers and used in a reverse line blot (RLB) hybridization assay enabling differentiation between *S*. *haematobium* and *S. bovis*. The assay was used to discriminate natural infections in wild caught *Bulinus globosus*.

**Conclusion:**

This research enabled the characterisation of species-specific DNA regions that enabled the design of species-specific oligonucleotides that can be used to rapidly differentiate between *S*. *haematobium* and *S. bovis* and also have the potential to aid the detection of natural hybridization between these two species.

## Background

Schistosomiasis is a parasitic disease prevalent in tropical and subtropical regions caused by blood flukes of the genus *Schistosoma* [1]. In 2012, about 42 million people were treated with the anti-schistosomal drug praziquantel, although in 2014 it was estimated that at least 258 million people required treatment [2]. Urogenital schistosomiasis in humans is caused by infection with *Schistosoma haematobium,* affecting ~112 million people in Africa and the Middle East, and another 436 million individuals are considered at risk of infection [1, 2]. The parasites reside in the blood vessels surrounding the bladder and eggs are released in the urine of humans. Infection takes place in freshwater that contains *Bulinus* species that serve as the snail intermediate hosts for the parasite [1].

*Schistosoma haematobium* is a member of a group of closely related schistosomes known as the *S*. *haematobium-*group schistosomes. The other species of this group all cause intestinal schistosomiasis. *Schistosoma intercalatum* and *S. guineensis* infect humans in isolated foci in central Africa, but how many people are infected and where transmission occurs is still unknown. *Schistosoma margrebowiei*, *S*. *leiperi*, *S*. *mattheei*, *S*. *curassoni* and *S. bovis* are parasites of domestic livestock and wild ungulates, mainly in Africa, with major veterinary and economic impact, but these species are not widely researched [3, 4]. All *S*. *haematobium* group species utilize *Bulinus* spp. for transmission. As the infective cercariae that emerge from the snail cannot be identified easily by morphological examination there is a need to provide reliable molecular markers to differentiate species. This is particularly important in endemic areas targeted for transmission control wherever the *S*. *haematobium-*group species co-exist [4, 5]. Transmission of *S. bovis* (a common cattle schistosome) most commonly overlaps with that of *S*. *haematobium*, and these two species are reported to be co-prevalent in many parts of Africa and the Middle East [6]. The other *S*. *haematobium-*group species also co-exist with *S. haematobium* in specific foci in various parts of Africa [6].

The need to identify which *S*. *haematobium* group species are transmitted by different *Bulinus* snails has led to the development of several DNA-based molecular methods for species-specific identification [7–9]. Molecular methods have included: Southern blot analysis [10], random DNA amplification [11], PCR-RFLP analysis of the ITS2 region of the ribosomal gene [12] and direct PCR amplification using species-specific primers targeting multi-copy gene regions such as the mitochondrial cytochrome oxidase subunit 1 [13] and amplification of genomic DNA repetitive fragments [7, 9]. A commonly used repetitive DNA segment identified in *S*. *haematobium* is the DraI repeat [14]. This tandemly arranged repeat sequence has been utilized in *Schistosoma* detection in *Bulinus* snails to identify patent and pre-patent infections and has enabled the screening of large populations of snails to evaluate the level of transmission [15]. The DraI repeat has also been used for the molecular diagnosis of *S*. *haematobium* infection by amplification of parasite DNA in human urine samples [16]. The DraI repeat offers high detection sensitivity but lacks specificity due to cross-amplification with the other *S*. *haematobium-*group species [14], hampering its diagnostic utility in sympatric areas and hybrid zones. Further studies have focused on finding DNA-based methodologies that are specific for the different *S*. *haematobium-*group species [9, 17]. Further repeat DNA sequences, the sh73bp and sh77bp, were targeted with an aim to find a species-specific DNA diagnostic marker for *S*. *haematobium*. An inter-repeat PCR, utilizing the DraI reverse primer and sh73bp forward primer produced a differential DNA banding pattern for *S*. *haematobium* (compared to that of *S. bovis*) providing a new one-step diagnostic PCR for the discrimination of *S*. *haematobium* from *S. bovis* [7, 9].

In this study we further elucidated the arrangement of the three repetitive DNA sequences, DraI, sh73bp, and sh77bp and their inter-repeat sequences, to further enable the differential identification of *S*. *haematobium* and *S. bovis*. The differences in the repeat arrangements were used to develop a new detection approach based on direct PCR and reverse line blot analysis that can be used for schistosome species-specific identification. The potential use of this new molecular diagnostic tool is discussed in relation to the monitoring of schistosomiasis transmission and also to help elucidate the natural and on-going hybridization of *S*. *haematobium* and *S. bovis* in sympatric areas of West Africa [18–20].

## Methods

### Origin of the schistosome and snails samples

For assay development, adult schistosome worms, *S*. *haematobium* (Mauritius NHM2695) and *S. bovis* (Senegal NHM196), were provided by the Schistosomiasis Collection at the Natural History Museum, London [21]. For assay testing, *Bulinus* snails were collected from Katchetu pond, an endemic area near the Mombasa-Nairobi highway in Katchetu village, Mazeras, Kenya. This pond has been known to contain significant numbers of *Bulinus globosus* snails infected with schistosomes. Goats, cattle, and humans frequently visit the pond for daily activities. Snails were collected by scooping, morphologically identified as *Bulinus* spp. and immediately preserved in 100% ethanol for molecular identification of *Schistosoma* infections.

### DNA extraction

DNA was extracted from the adult worms by digestion in 0.5 ml of lysis buffer (0.1 M EDTA, pH 8.0, 0.1 M Tris-HCl pH 7.5, 0.2 M NaCl, 1% SDS, 0.2% 2-mercaptoethanol and 100 μg Proteinase K) at 60 °C for 2 h followed by routine phenol extraction and ethanol precipitation method. Snails were removed from their shells and their whole bodies macerated in 0.5 ml of lysis buffer (0.1 M EDTA, pH 8.0, 0.1 M Tris-HCl pH 7.5, 0.2 M NaCl, 1% SDS, 0.2% 2-mercaptoethanol and 100 μg Proteinase K) and DNA extracted as above. The extracted DNA was suspended in TE buffer (1 mM Tris-HCl, 0.1 mM EDTA).

### Amplification and sequencing of the inter repeat region (sh73bp-DraI)

PCR reactions were carried out in a total volume of 25 μl using ready-mix PCR tubes (Syntezza, Jerusalem, Israel). Each reaction contained 20 pmoles of each forward (73 bp; 5′-CCT TGG TCA CGT GAT TTT C-3′) and reverse primer (DraI; 5′-TCA CAA CGA TAC GAC CAA CC-3′) and 5 μl of *S*. *haematobium* or *S. bovis* genomic DNA or snail-extracted DNA. The thermal cycling consisted of: 5 min at 95 °C, followed by 35 cycles of 30 s at 95 °C, 30 s at 58 °C, and 1 min at 72 °C. A final elongation step was carried out for 10 min at 72 °C. The amplicons produced for *S*. *haematobium* and *S. bovis* were separated by agarose gel electrophoresis. Multiple bands were produced and the smallest three bands of each PCR were individually cut out of the gel and purified using the Qiaquick gel extraction kit (Qiagen, Hamburg, Germany). Each purified band was inserted into pJET cloning vectors through blunt end ligation using the cloneJET PCR cloning kit (Thermo-Fisher, Grand Island, USA). DNA from the recombinant plasmids was purified using QIAprep spin miniprep Kit (Qiagen, Hamburg, Germany). The size of the inserts was checked by PCR using the pJET1.2 primers flanking the cloning site and PCR products were visualised and their length evaluated by agarose gel electrophoresis. The desired inserts were subsequently sequenced on an Automated DNA Sequencer (AB477) using T3 and T7 sequencing plasmid universal primers. The DNA sequences of the 3 different bands for both *S. bovis* and *S*. *haematobium* were edited and compared using multiple sequence alignment tools (ClustalW omega) provided by the European Bioinformatics Institute (EMBL-EPI, Cambridge, United Kingdom). For the reverse line blot analysis, two species-specific oligonucleotides were manually designed. Sh1 oligo1 and Sbov ologo1 are reverse complement to the sequence of the species-specific repeat linkers Sh64bp and Sb30bp, respectively. Although the complementary DNA sequence of Sh oligo2 is fully inserted in the DraI repeat sequence downstream to the repeat linker Sh64bp, it is still specific to the *S*. *haematobium* and the Sbov oligo 2 covers some nucleotides of the upstream sh73bp repeat (Table 1). The designed oligonucleotides were commercially ordered from Integrated DNA Technologies (Munich, Germany), with a covalent addition of amino group at their 5′-ends.

**Table 1:** Species-specific oligonucleotide sequences for *S*. *haematobium* and *S. bovis* that were covalently bound on BiodynC membranes used in the RLB analysis

Oligo number	*Schistosoma* species	Oligo name	Oligo-nucleotide sequence (5′ Amino modified)
1	*S*. *haematobium*	Sholigo1	CTAGAATAAGGGCTGTTCTA
2	*S*. *haematobium*	Sholigo2	ACTTTGCTTCGTCTGATATG
3	*S. bovis*	Sbovoligo1	AACCAAACAAGACGTACACA
4	*S*. *bovis*	Sbovoligo2	GACGTACACACCCAGCTCCA

### Reverse line blot analysis

The technique was performed as previously described [22, 23], involving two main steps, as follows:

#### Step 1: Binding of the oligonucleotides to the EDC membrane

Biodyne C (Pall Corporation, Pensacola, USA) nylon membranes were activated by 10% 1-ethyl-3-[3-dimethylaminopropyl] carbodiimide (EDC), and this was followed by covalent binding of 5′-end amino modified species-specific oligonucleotides (Table 1). The membrane with the bound oligonucleotides was cut into strips containing all the oligonucleotides used.

#### Step 2: Hybridization and colorimetric detection

Membrane strips were incubated in pre-hybridization solution (2× SSC, 0.1% SDS) for 30 min at 46 °C followed by hybridization with denaturized biotinylated PCR product produced using the 5′-biotin modified 73d forward primer and DraI reverse primer commercially ordered from Integrated DNA Technologies (Munich, Germany). Hybridization was performed at 46 °C for 1 h, followed by washing with 0.7× SSC, 0.1% SDS for 20 min. Hybridized biotinylated DNA was detected by incubating the strips in streptavidin-HRP (diluted in 2× SSC, 0.1% SDS) for 30 min at room temperature, and subsequently the strips were briefly washed 3 times in 2× SSC, 0.1% SDS. Colour developed after addition of a solution containing 0.1 mg/ml of 3,3′,5,5’tetramethylbezidine (TMB) (Sigma, USA), 0.003% H_2_O_2_ in 0.1 M sodium citrate (pH 5.0).

## Results

### DNA sequence analysis of the *S*. *haematobium* and *S. bovis* inter-repeat PCR amplification products (sh73bp-DraI)

**Fig. 1:**
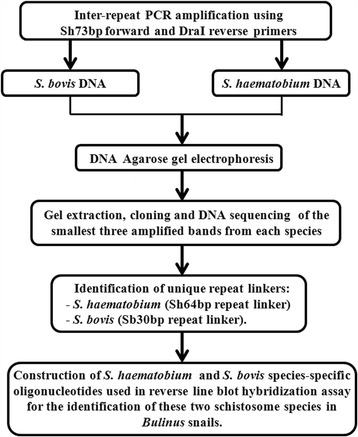
Flow diagram for the methodologies used in assessing the DNA sequence differences among *S*. *haematobium* and *S. bovis* amplified inter-repeats and in identification of unique sequences that were utilized in their detection and differentiation in *Bulinus* snail intermediate host

Figure 1 represents a flow diagram for the strategy used to show the DNA sequence differences among *S*. *haematobium* and *S. bovis* amplified inter-repeats and the development of a reverse line blot method for their detection and differentiation in *Bulinus* snail intermediate hosts. The smallest 3 bands resulting from the inter-repeat PCR were successfully cloned and sequenced.

**Fig. 2:**
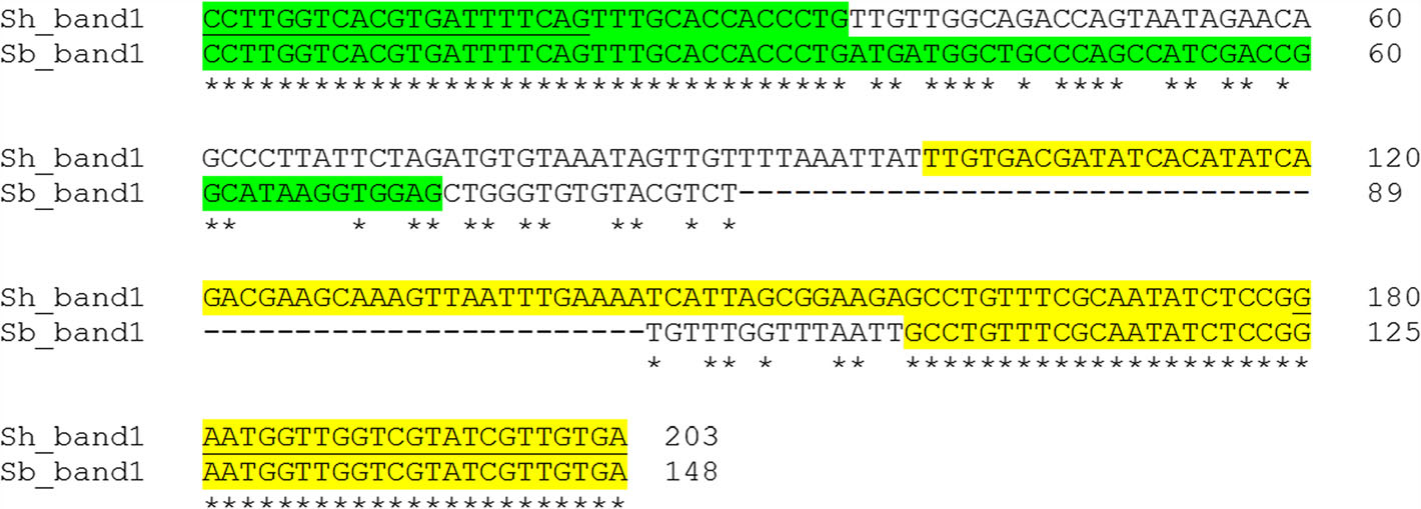
DNA sequence alignment of the smallest amplified inter-repeat band (using DraI reverse and 73 bp direct primers), when targeting *S*. *haematobium* genomic DNA (Sh_band1), and *S. bovis* genomic DNA (Sb_band1). *Yellow highlight* represents regions of DraI repeat, and *green highlight* represents sh73bp repeat regions. Un-colored regions are the unique sections for each species (the Sh64bp and Sb30bp repeat linkers). The *underlined sequences* indicate primers sites used in the PCR amplification

For *S*. *haematobium* the size of the smallest band was 203 bp, while that of *S. bovis* was 148 bp (Fig. 2). This 55 bp size difference could also be seen by agarose gel analysis, enabling visual differentiation between the two species. Figure 2 shows the DNA sequence of the *S*. *haematobium* inter-repeat first band compared to that of *S. bovis*. The *S*. *haematobium* band consists of a 35 bp section of the Sh73bp repeat found at the 5′ region, and 104 bp section of the DraI repeat found at the 3′ region connected by a unique 64 bp fragment which has been named the “Sh64 repeat linker”. The *S. bovis* band consisted of a complete unit of the Sh73bp repeat found at the 5′ region, and a shorter section of the DraI fragment (45 bp) located at the 3′ region connected by a unique 30 bp DNA fragment that has been named the “Sb30bp repeat linker” (Fig. 2).

**Fig. 3:**
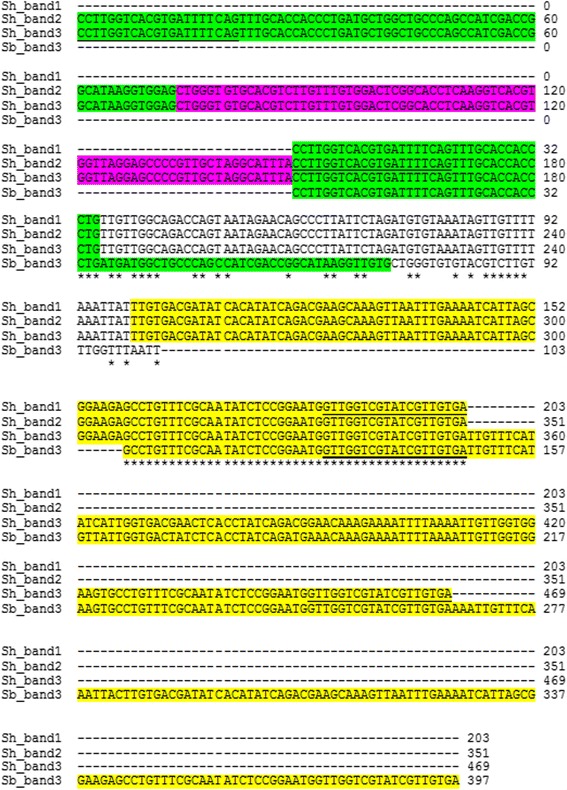
Comparison among DNA sequences of the three smallest *S*. *haematobium* PCR inter-repeat amplified bands (using DraI reverse and 73 bp direct primers) *vs* the DNA sequence of the third smallest amplified *S. bovis* band. *Yellow highlight* represents regions of DraI repeat, *green highlight* represents 73 bp repeat regions, *violet highlight* represents 77 bp repeat region. Un-colored regions are the repeat linkers in each species. The *underlined sequences* represent the locations of DraI reverse and 73 bp direct primers in the different amplified band units

The exact sizes of the smallest three amplified bands for *S*. *haematobium* were 203 bp, 351 bp, and 469 bp, while the corresponding sizes of the smallest three amplified bands of *S. bovis* were 148 bp, 296 bp and 387 bp. The amplified first and second bands within each species were completely contained within the third band. Figure 3 shows the alignments of the *S*. *haematobium* first three amplified bands as compared to the third band of *S. bovis*. The DNA sequence analysis of the *S*. *haematobium* second band shows a complete similarity to the smallest band, starting from the 5′ region, with an extra unit of the sh73bp repeat followed by a sh77bp repeat at the 3′-end. The third *S*. *haematobium* amplified band is differentiated from the second band by having an extra DraI repeat at the 3′-end, with an increment of 118 bp over the second band. Comparing the third amplified band from both *S*. *haematobium* and *S. bovis* shows that the smallest amplified band from each species is the core sequence, with additions of DraI units at the 3′-end, and sh77bp and sh73bp repeats appearing in an alternative manner at the 5′-end.

### Utilization *S*. *haematobium* and *S. bovis* sequence differences for species identification

**Fig. 4:**
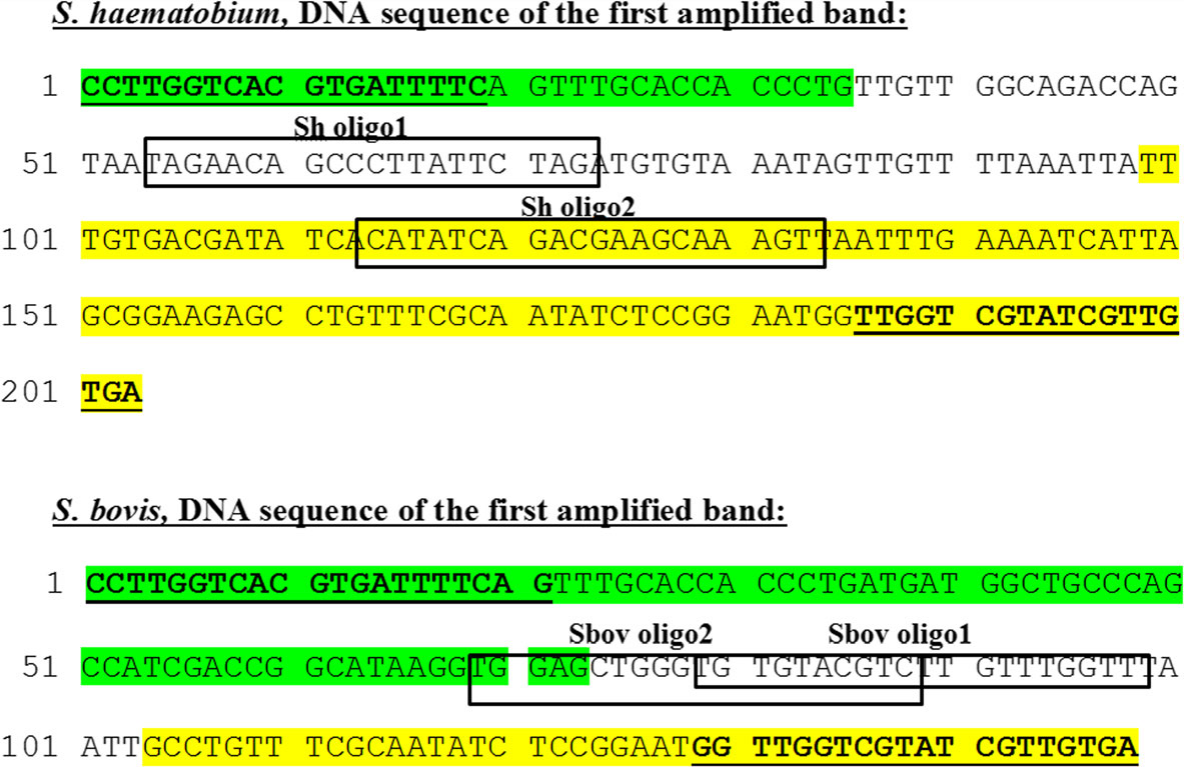
DNA sequence analysis of the smallest band of *S*. *haematobium* and *S. bovis* obtained by inter-repeat PCR amplification using DraI reverse and 73 bp forward primers. According to these DNA sequences, specific *S*. *haematobium* and *S. bovis* oligonucleotides (in *boxes*) were designed in unique regions, and these enabled differential identification. *Yellow highlight* represents regions of the DraI repeat, and *green highlight* represents the 73 bp repeat regions. Un-colored regions are the unique regions for each species. *Underlined bold* sequences are the DraI reverse and 73 bp forward primers

The new finding of a unique 64 bp (Sh64bp repeat linker) in the smallest inter-repeat PCR amplified bands from *S*. *haematobium* and of a unique 30 bp (Sb30bp repeat linker) in the smallest *S. bovis* amplified band (Fig. 3) allowed the design of species-specific oligonucleotides that could be used in reverse line blot analysis for the purpose of species differentiation. Based on this difference in DNA sequence, it was possible to find two oligonucleotides specific for *S*. *haematobium* and another set of two oligonucleotides that were specific for *S. bovis* (Table 1, Fig. 4).

### Using *S*. *haematobium* and *S. bovis* specific oligonucleotides in reverse line blot analysis (RLB) for species identification

**Fig. 5:**
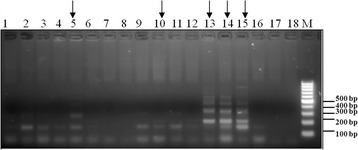
Banding patterns of inter-repeat PCR amplification (using DraI reverse and 73 bp direct primers) targeting total DNA extracted from *Bulinus globosus* (Lanes 1–15); Lane 16: from a previously tested negative snail; Lanes 17 and 18: negative control reactions without DNA. PCR was performed using biotinylated primers. Lanes that are marked by arrows represent the PCR products that were used for species identification by reverse line blot analysis

Production of biotinylated PCR amplicons of the inter-repeat region was achieved by using biotinylated DraI reverse primer and 73 bp forward primer targeting total genomic DNA extracted from *Bulinus globosus* snails that were collected from an area co-endemic by the two *Schistosoma* species. The currently analyzed snails were selected from previous experiments with known amplified banding pattern using DraI reverse primer and 73 bp forward primer and they represent different *S*. *haematobium* and *S. bovis* banding patterns. Agarose-gel electrophoresis analysis of the PCR products produced the known banding pattern for *S*. *haematobium* in some snails (Fig. 5, lane 13) and the known pattern for *S. bovis* in other snails (Fig. 5, lanes 2–5 and lanes 9–12). Two snails showed a mixed pattern of the two species, which exhibited as combined pattern of the *S*. *haematobium-* and *S. bovis-*specific bands (starting from 150 bp then 200 bp bands) (Fig. 5, lanes 14 and 15). In contrast to snail number 15, snail number 14 showed a stronger banding pattern for *S*. *haematobium* with a weaker smallest amplified band specific for *S. bovis*. This result could represent mixed infection by both *S*. *haematobium* and *S. bovis,* or the presence of hybrid schistosomes. The other examined snails did not show a positive amplification results (Fig. 5, lanes 1 and 6–8). Lane 16 is from a negative snail and lanes 17 and 18 represent negative control reactions without DNA.

**Fig. 6:**
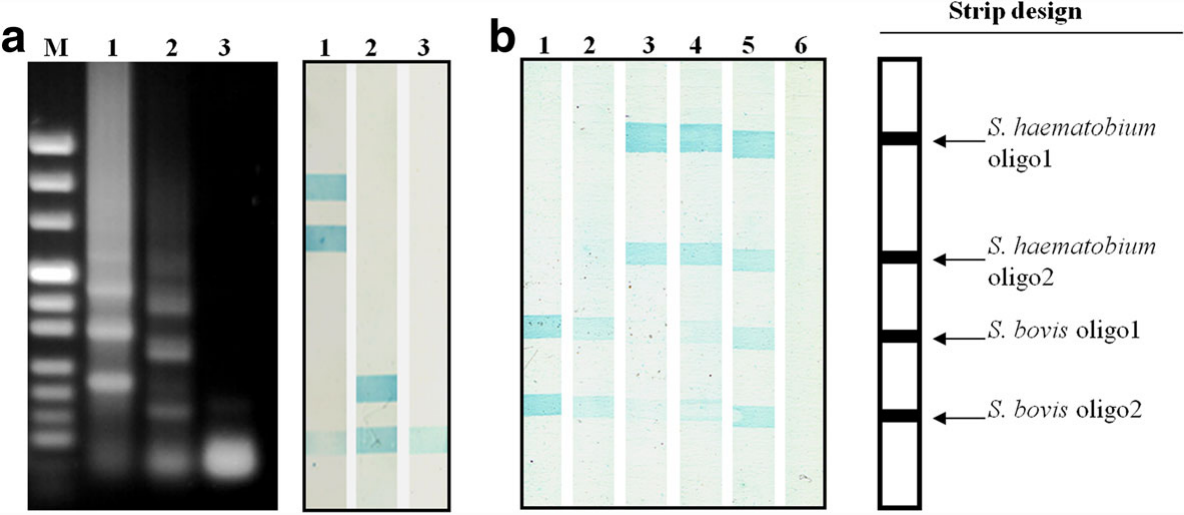
Reverse line blot (RLB) analysis. **a** Biotinylated PCR products targeting *S*. *haematobium* (Lane 1) and *S. bovis* (Lane 2) genomic DNA showing species specific PCR amplicons by agarose gel electrophoresis and their corresponding RLB analysis; Lane 3: negative control reaction without DNA. **b** The amplicons indicated by arrows in Fig. 5 (Lanes 5, 10, 13, 14 and 15) are shown in Lanes 1–5 of this figure. The “strip design” shows the covalent binding sites of the designed *S*. *haematobium* and *S. bovis* specific oligonucleotides to the Biodyn C membranes. About 15 μl of the amplified PCR products were used to hybridize with the indicated specific oligonucleotides. Strip number 6 represents a negative control

The amplified bands were then used in reverse hybridization procedure against oligonucleotides that were covalently bound to nylon membrane in a line format (RLB) (Fig. 6). The biotinylated PCR amplified products targeting *S*. *haematobium* and *S. bovis* genomic DNA were hybridized against the newly designed species-specific oligonucleotides and shown strong hybridization signals (Fig. 6a). In some reactions a weak signals corresponding to non-specific hybridization of *Schistosoma* biotinylated PCR amplified products to *S. bovis* oligo 2 was seen. This RLB hybridization procedure was also used to examine biotinylated amplified PCR products from selected snails shown in Fig. 5. The hybridization signals clearly identified the unique amplified bands of *S*. *haematobium* (Fig. 6b, lane 3) and *S. bovis* (Fig. 6b, lanes 1 and 2), that correspond to the PCR amplified products in Fig. 5 lane 13 and Fig. 5 lanes 5 and 10, respectively. It is clearly seen that the PCR amplified bands are fully reflected in the obtained hybridization signals as demonstrated by unique banding pattern and hybridization signals for the *S. bovis* and *S*. *haematobium* infected snails, (Fig. 5, lanes 5 and 10 and Fig. 5, lane 13, respectively). The PCR amplified banding pattern from snails 14 and 15 (Fig. 5) showed a mixed bands of both species and this was clearly seen also in the RLB hybridization results (Fig. 6, lanes 4 and 5). Furthermore, snail 14 that showed a stronger amplified band pattern for *S*. *haematobium* compared to *S. bovis*, produced a stronger hybridization signals with *S*. *haematobium* specific oligonucleotides compared to the signals with *S. bovis* specific oligonucleotides.

## Discussion

Previous studies have applied various molecular tools for differentiating *S*. *haematobium* from other related *Schistosoma* species [7–9, 12]. The inter-repeat (sh73-DraI) DNA sequence has provided a one-step PCR assay that enables differentiating *S*. *haematobium* mainly from *S. bovis* at the nuclear level [7, 9]. Here we clarify the unique arrangement of this inter-repeat region for *S*. *haematobium* and *S. bovis*, and describe the species-specific DNA sequences.

Three repeat regions have previously been described (DraI, sh73bp and sh77bp) that can be used as molecular diagnostic markers for members of the *S*. *haematobium* species group, but cannot distinguish between them [9, 14]. The DraI repeat is a direct repeat region of 120 bp producing a uniform ladder pattern whereas the sh73bp and sh77bp repetitive regions do not give a uniform banding pattern suggesting that there are other DNA segments intervening between any two constitutive sh73bp or sh77bp repeats [9, 14]. Amplification of the inter-repeat DNA region between the DraI and the sh73bp repeats gave a non-even ladder type banding pattern for both *S*. *haematobium* and *S. bovis*, with a unit band size that is larger by 55 bp increments. Here DNA sequence analysis of amplified bands from both *S*. *haematobium* and *S. bovis* revealed extra DNA segments or repeat linkers, Sh64bp and Sb30bp, respectively. These linking repetitive regions of the DraI tandem repeat with alternating repeats of sh73bp and sh77bp. Based on previous data from inter-repeat PCR amplification employing the DraI and the sh77bp repeats’ primers [9], it was found that the banding pattern is similar whether DraI reverse and 73 bp forward primers are utilized when targeting *S*. *haematobium* DNA, or when the DraI reverse and 77 bp forward primers are used for this purpose. This is consistent with the current finding regarding the sh73bp and sh77bp repeats and their association with DraI repeat. An additional conclusion that can be drawn from this information is that the alternative tandem arrangement of both sh73bp and sh77bp repeats is located at the 5′-end of DraI clusters, linked by the repeat linker, and there are no sh73bp or sh77bp repetitive sequences or clusters on the 3′-end of the DraI tandem repeats. This conclusion is supported by the finding that no successful PCR amplification with a ladder banding pattern can be obtained when employing the DraI forward primer with either the 73 bp or the 77 bp reverse primers [9].

The newly identified diverse regions (the Sh64bp and Sb30bp repeat linkers) were further developed into a one-step PCR assay with an aim to discriminate between *S*. *haematobium* and *S. bovis*. As a result, the DNA sequence differences were used to design species-specific oligonucleotides for *S*. *haematobium* and for *S. bovis* that can be used in reverse line blot hybridization for species identification. This strategy for detecting nucleotide differences between two PCR products was previously introduced for the identification of point mutations in genes causing genetic diseases [22]. It was also used for identification of differences in cytochrome *b* gene amplified by PCR from different mammalian species [23]. The major advantage in adding this strategy for differentiating between *S*. *haematobium* and *S. bovis* is to assure which amplified segments belongs to which species, and to avoid misclassification based on differences in band sizes obtained by PCR from these two species, or if and when other related species are involved. Moreover, these newly designed, species-specific oligonucleotides can be used in simple DNA oligochromatography following PCR amplification. The previously developed methods used in differentiating between *S*. *haematobium* and *S. bovis* are based on two steps that include PCR amplification of the target gene followed by DNA sequence analysis [12, 13]. The major potential of the currently developed PCR/RLB method used in discriminating between these two species is its suitability to be used in a mass screening of naturally infected snails, as well, its specificity that prevents DNA hybridization between *S*. *haematobium* and *S. bovis* species-specific oligonucleotides and genomic DNA of other related parasites.

Recent studies have highlighted the existence of introgressive hybridization between closely related schistosomes as a possible emerging public health problem that may pose a serious challenge for disease control and elimination programs [24, 25]. In this type of species hybridization, a single gene as well as complete chromosomal regions could be transferred from one species to another [26]. Webster et al. [20] has confirmed the occurrence of hybridization between *S*. *haematobium*, a human schistosome species, with two animal schistosomes, *S. bovis* and *S*. *curassoni*. Evidence for hybridization between *S*. *haematobium* and other animal species was provided based on production of different egg phenotypes, and DNA barcoding analysis of mitochondrial *cox1* gene and ITS regions in Senegal [20]. We believe that the differences in inter-repeat amplification (based on DraI and sh73bp repeats) between *S*. *haematobium* and other terminal-spined animal schistosomes could provide an additional tool for hybrid detection and monitoring of gene flow among these species. Taking into account that introgressive hybridization involves transfer of whole haploid chromosomes from one species into another, a mixed inter-repeat amplification pattern could be detected in this type of hybridization. In addition to this phenomenon, there are cumulative bioinformatics data based on DraI repeat DNA BLAST comparison in both *S*. *haematobium* and *S*. *mattheei* genomes, together with experimental data, pointing to the arrangement of DraI as cluster tandem repeats that are located close to other clusters of inverted tandem DraI repeats (unpublished data). This type of arrangement represents an excellent location for chromosomal crossover and an ultimate hybrid formation. The inter-repeat amplification targeting schistosome DNA within intermediate snail hosts shows a mixed banding pattern of both *S*. *haematobium* and *S. bovis* (Fig. 5, lanes 14 and 15). This type of amplification can be produced from mixed snail infections by the two parasites or by infection by one individual hybrid of these two parasites. This issue can be clarified only if a single shed cercariae released from a patent infected snail is subjected to PCR analysis utilizing the DraI reverse and 73 bp direct primers. Additionally, it would be possible to perform this type of PCR analysis on DNA from a single miracidium from a hatched egg isolated from an infected individual. The RLB test adds a new technological approach based on new sequence information for resolving this point. Further analysis of samples from this area of Kenya is warranted to detect whether hybridisation of *S*. *haematobium* and *S. bovis* is occurring, a phenomenon not yet investigated in East Africa.

Further DNA sequencing involving more schistosome samples from different geographical locations combined with bioinformatics analysis will be needed for determining the accurate clustering arrangement of DraI, sh73bp, and sh77bp repeats in the whole genome of *S*. *haematobium* and other related animal schistosomes. In addition, DNA sequencing of the amplified region using these primers directly from infected snails from different endemic regions will provide a more comprehensive view on newly emerging hybrid species, and could also provide better understanding of the biological significance of these repeats in the survival and evolution of *S*. *haematobium* group schistosomes.

## Conclusions

The current finding indicates the presence of DraI repetitive sequence as a cluster of units that are linked to alternating sh73 and sh77 repeats by repeat linkers. These have been named sh64bp in *S*. *haematobium* and sb30bp in *S. bovis*. DNA sequence differences in these two repeat linker regions were utilized in designing species-specific oligonucleotides for *S*. *haematobium* and *S. bovis.* These were then used in reverse line blot hybridization for the detection and differentiation of these species in naturally infected *B. globosus*. This new approach may aid the detection of the natural hybridization among *S*. *haematobium* and *S. bovis* in sympatric areas.

## Abbreviation

RLB: Reverse line blot
